# Effects of High Temperature and Drought Stress on the Expression of Gene Encoding Enzymes and the Activity of Key Enzymes Involved in Starch Biosynthesis in Wheat Grains

**DOI:** 10.3389/fpls.2019.01414

**Published:** 2019-11-12

**Authors:** Hongfang Lu, Yangyang Hu, Chenyang Wang, Weixing Liu, Geng Ma, Qiaoxia Han, Dongyun Ma

**Affiliations:** ^1^National Engineering Research Center for Wheat, College of Agriculture, Henan Agricultural University, Zhengzhou, Henan, China; ^2^State Key Laboratory of Wheat and Maize Crop Science, Henan Agricultural University, Zhengzhou, Henan, China; ^3^Henan Institute of Science and Technology for Development, Zhengzhou, Henan, China

**Keywords:** wheat, high temperature, drought stress, starch biosynthesis, enzyme activity, gene expression, starch accumulation

## Abstract

High temperature (HT) and drought stress (DS) play negative roles in wheat growth, and are two most important factors that limit grain yield. Starch, the main component of the wheat [][endosperm, accounts for 65–75% of grain weight, and is significantly influenced by environmental factors. To understand the effects of post-anthesis HT and DS on starch biosynthesis, we performed a pot experiment using wheat cultivar “Zhengmai 366” under field conditions combined with a climate-controlled greenhouse to simulate HT. There were two temperature regimes (optimum day/night temperatures of 25/15°C and high day/night temperatures of 32/22°C from 10 days after anthesis to maturity) accompanied by two water treatments (optimum of ∼75% relative soil water content, and a DS of ∼50% relative soil water content). Optimum temperature with optimum water treatment was the control (CK). We evaluated the expression patterns of 23 genes encoding six classes of enzymes involved in starch biosynthesis in wheat grains using real-time qPCR. HT, DS, and HT+DS treatments altered gene expression profiles. Compared to the CK, expression of 22 of the 23 genes was down regulated by HT, and only one gene (*ISA2*) was up-regulated by HT. Actually *ISA2* was the only gene up-regulated by all three stress treatments. The expression of 17 genes was up-regulated, while six genes, including granule-bound starch synthase (*GBSSI*)*, AGPS2*, *BEIII, PHOL, ISA1*, and *AGPL2*, were down-regulated by DS. Eleven genes were down-regulated and 12 were up-regulated by HT+DS. The activity of *ADP-Glc* pyrophosphorylase, starch synthases*, GBSS, SS, and* starch branching enzymes in the stress treatments (HT, DS, and HT+DS) often appeared to peak values in advance and declined significantly to be lower than that in the CK. The genes that coordinated participation in the enzymes formation can serve as an indicator of the enzymes activity potentially involved in starch biosynthesis. HT, DS, and HT+DS altered the timing of starch biosynthesis and also influenced the accumulation of amylose, amylopectin, total starch, and sucrose. Under HT, DS, and HT+DS, the key enzymes activity and their genes expression associated with the conversion of sucrose to starch, was reduced, which was the leading cause of the reductions in starch content. Our study provide further evidence about the effects of stress on starch biosynthesis in wheat, as well as a physiological understanding of the impact of post-anthesis heat and DS on starch accumulation and wheat grain yield.

## Introduction

Wheat (*Triticum aestivum* L.), grown in many different environments worldwide, is one of the major cereal crops and an important source of calories for human nutrition. Environmental constraints are major influence factors for wheat productivity in many regions. Wheat is particularly susceptible to temperatures above the range of 20–30°C, and mean temperatures during the wheat grain-filling stage often exceed the optimum in most wheat-growing regions, which is considered to be one of the most important factors limiting wheat grain yield (Skylas et al., 2002). Some wheat varieties can suffer yield loss of 10–15% with every temperature increase of 5°C (Burrell, 2003). Because of global warming, increase in average temperature of growing seasons will cause further reductions in wheat grain production (Asseng et al., 2011). Wheat is also very susceptible to water stress, particularly at the developmental stages of anthesis and grain-filling (Trnka et al., 2014). Drought stress accelerates grain filling and reduces total starch accumulation which is directly correlated with wheat productivity (He et al., 2012).

Starch, composed of amylose and amylopectin, is the main component in wheat endosperm. It accounts for 65–75% of grain weight, is the major determinant of grain yield. Starch deposition occurs synchronously with grain development in wheat (Altenbach et al., 2003). Starch accumulation peaks between 12 and 35 days after anthesis (DAA) during grain filling stage (Olsen et al., 1999; Olsen, 2001; Sabelli and Larkins, 2009; Sreenivasulu et al., 2010). Starch biosynthesis in wheat grains is a complex process that requires the coordinated participation of many enzymes. Amylose is synthesized by *ADP-Glc* pyrophosphorylase (*AGPase*) and granule-bound starch synthase (*GBSS*). Amylopectin is mainly synthesized by the coordinated actions of *AGPase*, starch synthases (*SS*s), starch branching enzymes (*SBE*s), and starch debranching enzymes (*DBE*s) (Hwang et al., 2005; Jeon et al., 2010). In addition, starch phosphorylase (*PHO*), metabolizing sucrose to the carbon precursors, is also needed for starch biosynthesis (Thitisaksakul et al., 2012). There are different isoforms of the enzymes involved in starch biosynthesis process. Such as, based on protein sequence comparisons, there are five main phylogenetic groups of *SS*: *SSI-SSIV* and *GBSS* (Leterrier et al., 2008). Both high temperature and drought stress can act either directly or indirectly to regulate a number of biochemical and physiological processes. In wheat, starch characteristics are much more sensitive to high temperature stress than protein synthesis (Bhullar and Jenner, 2005). Temperature influences the expression of genes that encode enzymes involved in starch biosynthesis (Dupont and Altenbach, 2003).

High temperatures and drought stress, as the major abiotic stress factors that affect wheat growth, development, and production (Noohi et al., 2009; Hasanuzzaman et al., 2012; Hossain et al., 2012; Bilal et al., 2015; Lobell et al., 2015), occur frequently and concurrently in many regions (Plaut et al., 2004; Rizhsky et al., 2004; Hirschi et al., 2011). A better understanding of the combined effects of high temperature and drought stress is important (Barnabas et al., 2008) when considering future climate scenarios in which seasonal changes in temperature and drought are projected (IPCC, 2007). The study of how starch biosynthesis enzymes respond to high temperature and drought stress is crucial for wheat genetic improvement to ensure high yield under adverse conditions. However, the mechanisms underlying the effects of high temperature and water deficit on starch biosynthesis and accumulation in wheat grain endosperm are not well understood. Only a few genes or enzymes involved in starch biosynthesis were evaluated in a previous report (Thitisaksakul et al., 2012), and the expression of many other genes in wheat grains, such as *SSIIb, SSIIc, SSIIIb, SSIV, BEIIb, BEIII, GBSSII, ISAI, ISA2, PUL, PHOH,* and *PHOL*, have not yet been examined under conditions of high temperature and water deficit. Furthermore, most studies examine the effects of either high temperature or drought stress seperately, thus give insufficient knowledge of the combined effects of high temperature and drought stress. Therefore, in this study, we analyzed the relative transcription of 23 isozyme genes encoding enzymes involved in starch biosynthesis in wheat grains using real-time qPCR. We also evaluated the activities of starch biosynthetic enzymes, and the accumulation of starch and sucrose. Furthermore, we simulated the starch biosynthesis process in response to high temperature and drought stress. The expected results will enable a better understanding of the influence of high temperature, drought stress, and their interaction on starch biosynthesis, and will also extend our knowledge on the impact of post-anthesis heat and drought stress on starch synthesis and grain yield in wheat.

## Materials and Methods

### Plant Material, Stress Treatments, and Sample Collection

Seeds of “Zhengmai 366,” a widely cultivated local wheat variety, were obtained from the Henan Academy of Agricultural Sciences. An experiment combining pot culture with a climate-controlled greenhouse to simulate high temperature was conducted at the farm of the Scientific and Educational Station of Henan Agricultural University, Zhengzhou, China (34°N, 114°E) during the 2015–2016 and 2016–2017 growing seasons. Seeds were sown in 24 cm-diameter pots containing 10 kg loam soil. The soil was collected from the top 20 cm soil layer at the experimental site. The total N, rapidly available phosphorus, and potassium were 1.43, 68.14, and 204.87 mg/kg, respectively. Fertilizer was applied at a rate of 1.15 g N, 1.35 g P_2_O_5_, and 1.15 g K_2_O per pot before sowing. Pots were buried in the field, keeping the soil surface inside the pot at the same level as that of the field. The trial was arranged in a randomized complete block design with three replicates. The plants were thinned to 12 seedlings per pot at the three-leaf stage. An additional 1.1 g N per pot was then added at the stem elongation stage.

The spikes were tagged for sampling when the anthers had extruded from the florets in 50% of the tillers in each pot. The plants in pots were grown under field conditions until they were transferred to the climate chamber for high temperature treatment beginning at 10 days after anthesis (10 DAA). In the controlled climate chambers, two temperature regimes consisting of optimum day/night temperatures of 25/15°C and high day/night temperatures of 32/22°C were accompanied by two water treatments; an optimum water treatment with ∼75% of the relative soil water content, and a mild drought stress treatment with ∼50% of the relative soil water content, was set to wheat maturity. The optimum day/night temperature combined with the optimum water treatment was the control (CK), and the plants were irrigated regularly to maintain the soil relative water content at ∼75%. High day/night temperature with optimum water treatment was defined as the high temperature (HT) treatment. For the drought stress (DS) treatment, the water content was controlled from ∼3 DAA prior to transfer to the climate chambers to ensure that the soil relative water content was maintained at the objective level of ∼50%. The soil water content was determined by weighing the pots, combined with time domain reflectometry measurements (TDR300, Spectrum). The treatment consisting of high day/night temperature and DS was defined as the combined HT and drought stress (HT+DS) treatment.

Tagged heads were initially collected before plants were transferred to the climate chamber. Sampling was then performed every 4 days until seed maturity. One portion of the grains sampled were frozen immediately in liquid nitrogen for 30 min and then stored at °80°C for RNA extraction and cDNA synthesis as well as for determination of the activity of starch biosynthesis enzymes, while another portion of the grains sampled were dried to constant weight for measurement of sucrose, soluble sugar, and starch contents as well as for determination of the starch accumulation rate. All the determination was in triplicate for each biological replicate and each sampling date contained three biological replicates.

### Ribonucleic Acid Extraction and Complementary Deoxyribonucleic Acid Synthesis

RNA was extracted from wheat grains at each sampled time point during grain development. Total RNA from grain endosperm was extracted using a test kit from Takara and purified with gDNA Eraser (Takara) to remove contaminating genomic DNA. cDNA was synthesized from 2 µg of total RNA using the ThermoScript Reverse Transcriptase Reagent Kit (Takara). The integrity of the RNA samples was examined by gel electrophoresis.

### Quantitative Real-Time Reverse Transcriptase Polymerase Chain Reaction

Expression profiles of the wheat genes were determined by quantitative real-time PCR analysis. cDNAs for all of studied genes were amplified using primers designed from known wheat nucleotide sequences (**Supplementary Table 1**). The wheat β-actin gene (GeneBank accession number AB181991) was used as the reference gene. Amplification products for each primer set were cloned and sequenced to ensure the gene identities. Quantitative real-time RT-PCR analysis was performed on an Applied Biosystems LightCycler 7300 system using the SYBR Premix Ex Taq II (Perfect Real-Time) Kit (Takara). The cycling conditions were as follows: 95°C for 5 min, followed by 40 cycles of 95°C for 5 s and the optimal annealing temperature of 60°C for 30 s. The specificity of the PCR amplification in each reaction was checked with a melting curve analysis (from 55 to 94°C) following the final cycle of amplification.

### Determination of the Activities of Enzymes Involved in Starch Biosynthesis

We assayed the activities of five starch biosynthesis enzymes, including starch synthase (*SS*), *GBSS*, soluble starch synthase (*SSS*), *AGPase*, and starch branching enzyme (*SBE*), respectively, using the reagent kits *SS-2-Y*, *GBSS-2A-Y*, *SSS-S-Y*, *AGP-2A-Y*, and *SBE-2-Y* obtained from Su Zhou Keming Biotechnology Incorporated. The activities of *GBSS*, *SSS*, and *AGPase* were determined as nmol min^–1^ of *NADPH* produced g^–1^ grain *FW*; *SS* activity was measured by quantifying the production of sucrose from fructose and glucose (µg min^–1^ g^–1^ grain FW); and *SBE* activity was determined as the percentage decrease in absorbance at 660 nm of that decrease of iodine blue value as an enzyme activity unit.

### Determination of Soluble Sugar and Sucrose Contents

The contents of soluble sugars and sucrose in wheat grains were determined using the anthrone colorimetric and resorcinol colorimetric methods as described by Li (2000).

### Determination of Amylose and Amylopectin Contents

The amylose and amylopectin contents in wheat grains were determined with a coupled spectrophotometric assay as described by He (1985).

### Determination of Starch Accumulation Rate

Determination of starch accumulation rate was referenced to the method of Wang et al. (2011). The rate of starch accumulation is shown as the increase in milligrams of starch accumulation per wheat grain per day and was calculated by dividing the difference between the starch accumulation per wheat grain before and after adjacent sampling by the number of intervening days.

### Data Analysis

Data from three biological replicates in the experiment were statistically analyzed for variance and correlation using SPSS 21.0. The line graphs were prepared and edited with SigmaPlot 13.0. Venn diagrams of gene expression were obtained using the Bioinformatics & Evolutionary Genomics online analysis software (http://bioinformatics.psb.ugent.be/webtools/Venn/).

## Results

### The Different Expression Profile of Genes Encoding Enzymes Involved in Starch Biosynthesis Under High Temperature and Drought Stress

The expression patterns of 23 genes encoding six classes of enzymes involved in starch biosynthesis in wheat grains were all evaluated by real-time PCR method. *AGPase* consists of four isoforms encoded by five isozyme genes, including *AGPL1*, *AGPL2*, *AGPS1-a*, *AGPS1-b*, and *AGPS2*; *SS* consists of four isoforms encoded by seven genes in wheat, including *SSI, SSIIa, SSIIb, SSIIc, SSIIIa, SSIIIb*, and *SSIV*; *GBSS* is encoded by two isozyme genes, *GBSSI* and *GBSSII*; *SBE* consists of three isoforms encoded by four genes in wheat, including *BEI, BEIIa, BEIIb*, and *BEIII*; *DBE* consists of two isoforms encoded by three genes in wheat, including *ISA1*, *ISA2*, and *PUL*; *PHO* consists of two isoforms encoded by two genes in wheat, including *PHOL* and *PHOH* (**Supplementary Table 1**).

**Figure 1:**
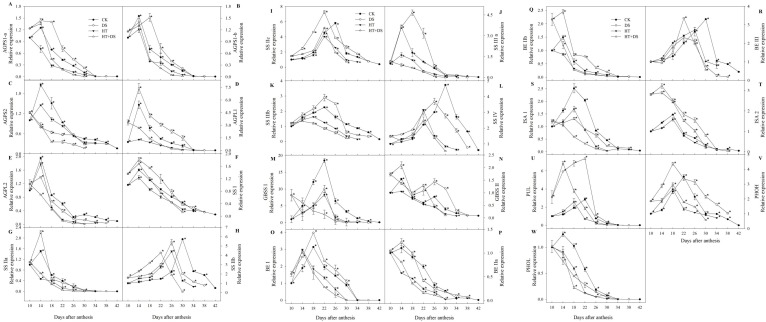
Expression profiles in wheat grain endosperm of 23 genes **(A–W)** encoding enzymes involved in starch biosynthesis from 10 to 42 days after anthesis in response to temperature and drought stress treatments. Filled circles, control (CK); open circles, drought treatment (DS); filled triangles, high temperature treatment (HT); open triangles, HT+DS treatment. Asterisks (*) indicate significant differences (p 0.05) between the individual treatments (CK, DS, HT, HT+DS).

In the CK, the expression of 23 genes all displayed a unimodal curve with the peak times varying for the different genes (**Figure 1**). The genes: *AGPL1*, *AGPL2*, *AGPS1-a*, *AGPS1-b*, and *AGPS2*; *SSI*, *SSIIa*, and *SSIIIa*; *GBSSII*; *BEIIb* and *BEIIa*; *PHOL,* showed expression peaks at 14 DAA. The expression of *BEI*, *ISA1*, *ISA2*, and *SSIIIb*, *GBSSI*, *PUL*, *PHOH* peaked at 18DAA and 22 DAA, respectively. While *SSIIb*, *SSIIc* expression peaked at 26 DAA, and *SSIV, BEIII* expression peaked at 30 DAA. Under HT, DS, and DS+HT, the peak times of the genes expression were modified and led to the change of the expression patterns. Compared with the CK, the genes (*SSIIb, SSIIc, SSIV*, *BEIII*, *ISA2*, and *PHOH*) expression in DS treatment peaked by 4 days (apart from *BEIII* by 8 days) in advance, while *AGPS1-b* and *SSIIIa* expression peaked by 4 days late. For the HT, HT+DS treatments, the genes expression, including *SSIIb, SSIIc, SSIIIb*, *SSIV*, *BEI*, *BEIII*, *PUL*, and *PHOH*, peaked by 4 or 8 days earlier than the CK. However, the expression of genes (*AGPS1-a*, *AGPS2*, *SSIIIa, BEIIa, BEIIb,* and *PHOL)* in HT treatment, and the expression of genes (*AGPL1*, *AGPL2*, *AGPS2*, *SSIIa*, *SSIIIa, SSIIIb*, *GBSSI, GBSSII*, *BEIIa, BEIIb*, *PHOL*) in HT+DS treatment, all had the maximum values at 10 DAA, and showed gradually declining trend during grain filling stage.

The results showed that, during the grain filling stage, compared with the CK, DS decreased the relative expression of *AGPS2*, *AGPL2, GBSSI*, *ISA1*, *PHOL* while significantly increased the expression of *AGPS1-a*, *AGPS1-b*, *AGPL1*, *BEI*, *BEIIb*, *ISA2*, *PUL*, *PHOH*; HT significantly reduced the expression of *AGPL1*, *AGPL2*, *AGPS1-a*, *AGPS1-b*, *AGPS2, SSI*, *SSIIa, SSIIc, SSIIIa, SSIIIb, ISA1*, *ISA2*, *PHOL;* HT+DS significantly reduced the expression of *AGPL1*, *AGPL2*, *AGPS1-a*, *AGPS1-b*, *AGPS2, SSIIa, SSIIIa, SSIIIb, ISA1, PHOL,* while only led to the increase of *ISA2* expression (**Figure 1**). For *GBSSII* in DS treatment, *GBSSII*, *BEI*, *BEIIa,* and *BEIIb* in HT and HT treatments, the genes expression was higher than that in the CK prior to 14 DAA, after which significantly declined and was much lower than that in the CK. While the expression of *SSIIa*, *BEIIa* in DS treatment, *PUL, PHOH* in HT and HT+DS treatments, *SSI* in HT+DS treatment, was higher than that in the CK prior to 18 DAA. Compared with the CK, DS, and HT+DS enhanced the expression of *SSIV*, *BEIII* before 22 DAA, after which the genes expression significantly declined to the lower levels. While the expression of *SSIIb, SSIIIa, SSIIIb*, and *SSIV* in DS treatment, *SSIIIb* and *BEIII* in HT treatment, *SSIIIb* in HT+DS treatment, increased before 26 DAA, after which it significantly decreased and was lower than the CK. In a word, HT and DS ultimately all led to the decline of the genes expression, and the negative effect of HT on the genes expression was greater than DS.

### High Temperature and Drought Stress Affect the Expression Level of Genes Encoding Enzymes Involved in Starch Biosynthesis

**Figure 2:**
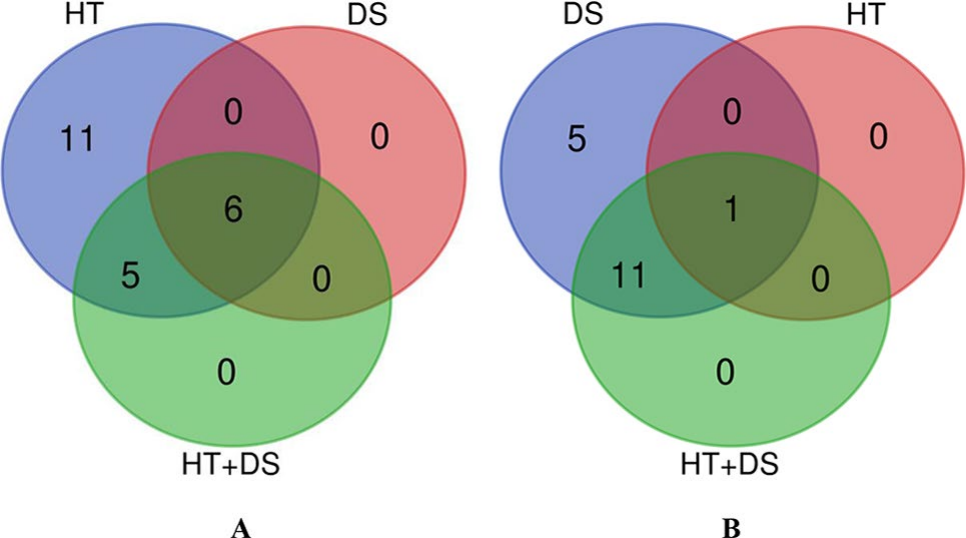
Venn diagrams of the relative changes in expression for 23 genes encoding enzymes involved in starch biosynthesis in the high temperature (HT), drought stress (DS), and HT+DS treatments compared with the CK. **(A)** Down-regulated gene expression; **(B)** up-regulated gene expression.

According to the difference values in the average relative expression quantity at all sampling points between the three stress treatments (HT, DS, and HT+DS) and the CK, the positive and negative values represent up-regulated and down-regulated gene expression, respectively. Of the 23 genes examined, relative expression in 22 was down-regulated by HT (**Figure 2A**), and only one gene (*ISA2*) showed up-regulated expression; *ISA2* was the only gene in which expression was up-regulated by the HT, DS, and HT+DS treatments (**Figure 2B**). In the DS treatment, 17 of the 23 genes showed up-regulated expression (**Figure 2B**), while the expression of six genes (*GBSSI*, *AGPS2*, *BEIII*, *PHOL*, *ISA1*, and *AGPL2*) was down-regulated (**Figure 2A**). We also found that the relative expression of 11 of the 23 genes was down-regulated and 12 was up-regulated by the HT+DS treatment. HT negatively influenced the expression of most of the 23 genes, while DS had the opposite effect. The result of the HT+DS treatment was not consistent with that of the DS or HT treatment, possibly implying that there is either a synergistic or an antagonistic effect on gene expression when the HT and DS conditions occur simultaneously.

### Dynamic Changes in the Activities of Key Enzymes Related to Starch Biosynthesis in Response to High Temperature and Drought Stress

The results from the 2015–2016 growing season were consistent with the 2016–2017 results. The following analysis is based on data from the 2016–2017 growing season.

**Figure 3:**
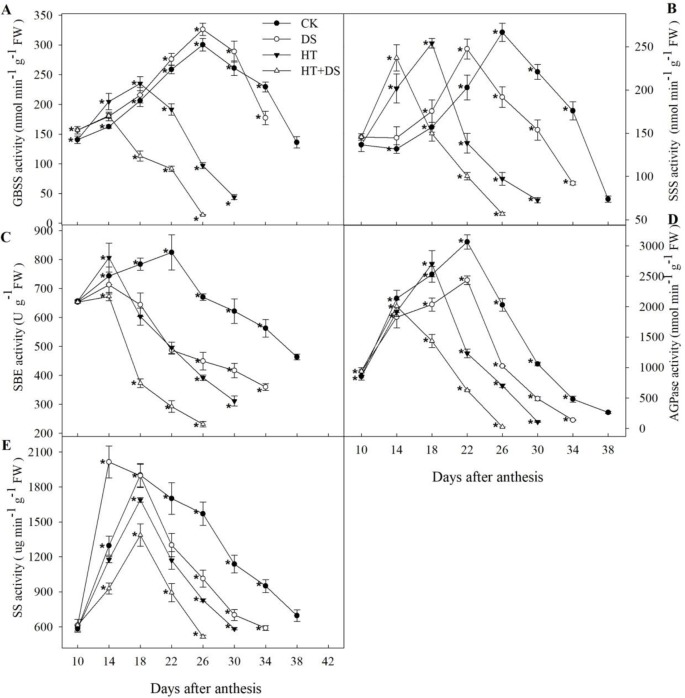
Dynamic changes in the activities of enzymes **(A–E)** related to starch biosynthesis in wheat endosperm from 10 to 42 days after anthesis in response to temperature and drought stress treatments. Filled circles, control (CK); open circles, drought treatment (DS); filled triangles, high temperature treatment (HT); open triangles, HT+DS treatment. Asterisks (*) indicate significant differences (p 0.05) between the individual treatments (CK, DS, HT, HT+DS).

The dynamic change of the starch biosynthetic enzymes was unimodal curve trend in different treatments (**Figure 3**). Compared with the CK, DS led to the activity of SS, SSS, and SBE peaking by 4 and 8 days in advance, respectively. The peak time of *AGPase* activity in HT treatment was 4 days earlier than that in the CK, while the activity of *SSS, GBSS,* and *SBE* peaked by 8 days in advance. In the HT+DS treatment, the activity of *AGPase*, *SBE,* and *SSS, GBSS* peaked by 8 and 12 days earlier, respectively, than that in the CK. The enzymes activity in HT+DS treatment peaked the earliest implying the superimposed effect between HT and DS.

Compared with the CK, DS treatment increased *GBSS* activity in general, while *GBSS* activity in the HT and HT+DS treatments was raised prior to the peak times at 18 and 14 DAA, respectively, after which sharply declined and was significantly lower than that in the CK (**Figure 3A**). *SSS* activity in the different treatments varied mainly with respect to the timing of the peak activity (**Figure 3B**). Peak activities were very similar for the four treatments, and peak times were 14 DAA for HT+DS, 18 DAA for HT, 22 DAA for DS, and 26 DAA for the CK. In all treatments, *SSS* activity declined steadily over 12 days after the peak to a very low value, and simultaneously HT, HT+DS treatments significantly reduced the SSS activity. HT, DS, and HT+DS all led to lower activity of *AGPase* and *SBE* than the CK in general during the grain filling (**Figures 3C, D**). The activity of *SS* in DS treatment was higher than that in the CK before the peak time at 14 DAA, and then rapidly declined to lower values than that in the CK, while HT and HT+DS significantly reduced the *SS* activity during the grain filling stage (**Figure 3E**). Compared with the CK, the activity of *SBE* was reduced about by 31, 23, and 41%, and the activity of *SS* was reduced about by 23, 19, and 32% in HT, DS, and HT+DS treatments, respectively. The HT+DS treatment declined by about two-fold compared with DS treatment. We found that the HT+DS treatment showed the greatest decline of the enzymes activity in all the treatments, and the negative effect of HT on the enzymes activity was greater than DS.

### Dynamic Changes in the Sucrose and Soluble Sugar Contents in Wheat Grains

**Figure 4:**
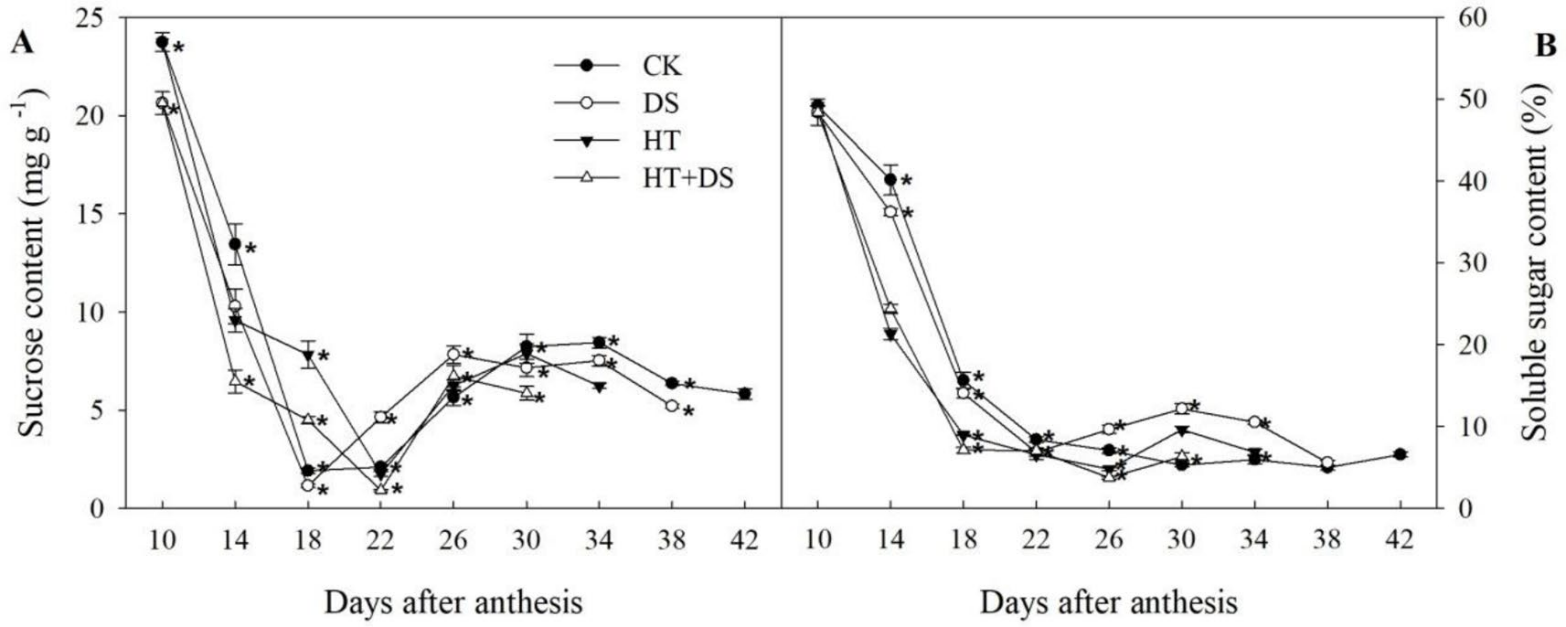
Dynamic changes in the contents of sucrose **(A)** and soluble sugars **(B)** in wheat grains from 10 to 42 days after anthesis in response to temperature and drought stress treatments. Filled circles, control (CK); open circles, drought treatment (DS); filled triangles, high temperature treatment (HT); open triangles, HT+DS treatment. Asterisks (*) indicate significant differences (p 0.05) between the individual treatments (CK, DS, HT, HT+DS).

HT and DS influences the dynamic changes in the sucrose and soluble sugar contents during the grain filling stage (**Figure 4**). In the stress treatments (DS, HT, and HT+DS), the sucrose content was lower than that in the CK before 18 DAA, and then higher, while sharply declined after 26 DAA and was significantly lower than that in the CK finally. The soluble sugar contents in the DS, HT, and HT+DS treatments were lower than that in the CK in general before 26 DAA, and then there were varying degrees of increase. While the soluble sugar contents of different treatments showed no significant differences in the end.

### Dynamic Changes in the Starch Accumulation Rate and the Amylose, Amylopectin, and Total Starch Contents

**Figure 5:**
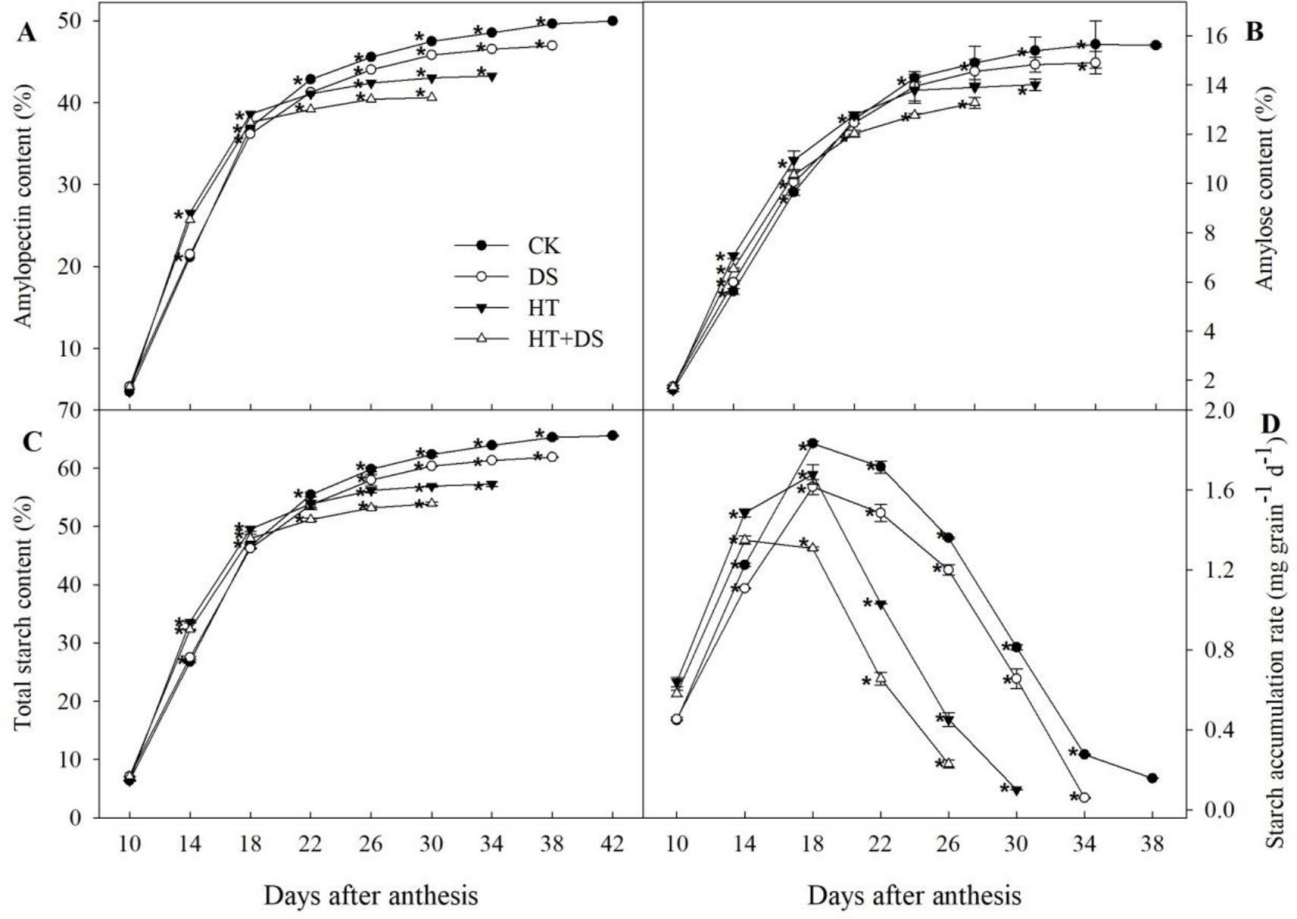
Dynamic changes in amylopectin **(A)**, amylose **(B)**, total starch content **(C)**, and the starch accumulation rate **(D)** in wheat grains in response to temperature and drought stress treatments. Filled circles, control (CK); open circles, drought treatment (DS); filled triangles, high temperature treatment (HT); open triangles, HT+DS treatment. Grains were assayed from 10 to 42 days after anthesis **(A** and **C)**, and from 10 to 38 days after anthesis **(B** and **D)**. Asterisks (*) indicate significant differences (p 0.05) between the individual treatments (CK, DS, HT, HT+DS).

Exposure to the DS, HT, and HT+DS conditions, wheat was premature earlier by 4, 8, and 12 days, respectively, than the CK (**Figure 5**). Dynamic changes in the starch accumulation rate in the different treatments followed a unimodal curve (**Figure 5D**). The starch accumulation rate in the HT+DS treatment appeared to peak at 14 DAA, while the CK, DS, and HT treatments all peaked at 18 DAA. The starch accumulation rate in the stress treatments (DS, HT, and HT+DS) was all significantly lower than that in the CK after 18 DAA. The accumulation of amylose, amylopectin, and total starch showed similar trends in the different treatments. In the HT, DS, and HT+DS treatments, the amylose (**Figure 5B**), amylopectin (**Figure 5A**), and total starch (**Figure 5C**) contents were significantly less than that in the CK after 18 DAA, which matches the timing of the decline in the starch accumulation rate.

### Effects of High Temperature and Drought Stress on Starch Content at Maturity

Adverse conditions significantly decreased the amylose, amylopectin, and total starch content in mature wheat grains, while the ratio of amylose to amylopectin was not significantly influenced (**Table 1**). The results from the 2015–2016 growing season was consistent with the results from 2016 to 2017. Compared with the CK (2016-2017), the amylose content was reduced by 10.2, 4.2, and 14.9%, the amylopectin content was reduced by 13.4, 6.0, and 18.7%, and the total starch content was reduced by 12.7, 5.6, and 17.8% in the HT, DS, and HT+DS treatments, respectively. The HT+DS made the greatest decline and by more than three-fold compared with DS. In a word, high temperature (HT, HT+DS) made greater effect on starch content than DS, and DS aggravated the negative effect of HT indicating a superimposed effect between HT and DS.

**Table 1:** Total starch, amylose, and amylopectin contents in mature wheat grains for the three stress treatments.

Treatment	2015–2016 growing season				2016–2017 growing season			
	Amylose (%)	Amylopectin (%)	Total starch (%)	Amylose/amylopectin	Amylose (%)	Amylopectin (%)	Total starch (%)	Amylose/amylopectin
CK	15.9 a	50.8 a	66.7 a	0.31 c	15.6 a	50.0 a	65.6 a	0.3 a
DS	15.3 b	47.1 b	62.4 b	0.32 b	15.0 b	46.9 b	61.9 b	0.3 a
HT	14.2 c	43.8 c	58.1 c	0.33 b	14.0 c	43.2 c	57.3 c	0.3 a
HT+DS	13.6 d	40.8 d	54.4 d	0.33 a	13.3 d	40.6 d	53.9 d	0.3 a

Different lowercase letters in each column indicate the significant differences (P < 0.05).

### The Effect of the Interaction Between High Temperature and Drought Stress on the Activities of Key Enzymes Related to Starch Biosynthesis

We performed an analysis of variance, based on the different growing seasons and measurement periods, to further explore the effects of the interaction between HT and DS. This analysis showed that HT significantly affected the enzyme activities measured in the experiment except for the *SBE* and *AGPase* activities at 14 DAA, and that DS significantly influenced the enzyme activities except for *GBSS* activity at 14 DAA (**Table 2**). A significant interaction effect was found for HT and DS.

**Table 2:** Variance analysis of the interaction of high temperature and drought stress and the activities of key starch biosynthesis enzymes.

Enzyme	Source	2015–2016 growing season				2016–2017 growing season			
		14DAA	18DAA	22DAA	26DAA	14DAA	18DAA	22DAA	26DAA
GBSS	HT	22.8**	12.7**	1290.8**	4956.8**	12.6**	19.8**	728.5**	3297.7**
	DS	1.5	142.6**	139.3**	5.0	0.3	46.6**	78.8**	40.5**
	DS×HT	8.3*	301.4**	199.3**	134.1**	11.4**	63.1**	158.1**	147.6**
SSS	HT	319.1**	213.3**	216.0**	344.1**	113.7**	50.0**	277.7**	902.8**
	DS	9.1*	91.6**	42.9**	49.2**	10.0*	72.5**	0.3	130.2**
	DS×HT	0.0	168.7**	50.4**	10.8*	2.2	148.7**	42.7**	11.6**
SBE	HT	0.6	141.4**	531.3**	116.6**	0.3	190.5**	174.8**	621.4**
	DS	46.5**	114.3**	560.8**	84.4**	17.9**	127.6**	186.2**	368.0**
	DS×HT	20.9**	13.2**	99.8**	0.7	7.0**	7.6*	11.5**	9.2*
AGPase	HT	1.5	10.0*	433.4**	358.7**	0.3	6.3*	1682.4**	1390.1**
	DS	6.0*	98.0**	216.2**	223.9**	2.2	107.6**	194.6**	726.5**
	DS×HT	15.0**	20.7**	3.3	26.2**	7.1*	20.7**	0.1	27.4**
SS	HT	174.0**	40.1**	449.2**	378.9**	154.8**	52.9**	66.0**	297.3**
	DS	13.3**	3.8	25.2**	51.0**	23.6**	9.6*	34.4**	146.4**
	DS×HT	43.5**	5.6*	0.2	20.4**	99.2**	9.0*	1.1	11.5**

Asterisks indicate significant differences at p< 0.05 (*) and p < 0.01 (**). DAA, days after anthesis.

### Correlation Analysis of Starch Biosynthesis Enzyme Activities With the Expression of Their Isozyme Genes

The expression of isozyme genes is obviously related to the activities of the enzymes they encode during different phases of grain filling, especially at the late stage (**Table 3**). The activities of *AGPase*, *SSS*, *GBSS*, and *SBE* showed significant correlations (p < 0.05 or p < 0.01) with the expression of their isozyme genes as a whole at 22–26 DAA.

**Table 3:** Correlation analysis between the activities of key enzymes related to starch biosynthesis and the relative expression of the genes that encode them.

Gene name	Enzyme activity	Days after anthesis			
		14 d	18 d	22 d	26 d
AGPS1-a	AGPase	−0.051	−0.153	0.734**	0.599*
AGPS1−b	AGPase	0.356	−0.103	0.727**	0.724**
AGPS2	AGPase	0.581*	0.789**	0.631*	0.747**
AGPL1	AGPase	−0.120	−0.051	0.598*	0.360
AGPL2	AGPase	0.246	0.412	0.964**	0.945**
SSI	SS	−0.114	−0.618*	0.795**	0.712**
SSIIa	SS	−0.779**	−0.104	0.776**	0.828**
SSIIb	SS	0.729**	−0.410	−0.568	0.076
SSIIc	SS	−0.123	−0.287	0.604*	0.984**
SSIIIa	SS	−0948**	−0.084	0.866**	0.855**
SSIIIb	SS	−0.822**	0.023	0.989**	0.714**
SSIV	SS	−0.633*	−0.484	0.096	0.827**
GBSS I	GBSS	−0.454	0.338	0.803**	0.515
GBSSII	GBSS	−0.419	−0.706*	−0.441	0.847**
BEI	SBE	−0.015	0.651*	0.620*	0.711**
BEIIa	SBE	−0.329	0.776**	0.921**	0.898**
BEIIb	SBE	−0.340	0.568	0.052	0.282
BEIII	SBE	0.601*	−0.542	−0.328	0.412

Asterisks indicate significant correlations at p< 0.05 (*) and p < 0.01 (**).

### Correlation Analysis of Starch Biosynthesis Isozyme Gene Expression With the Dynamic Accumulation of Soluble Sugars, Sucrose, and Starch

The expression of genes for isozymes involved in starch biosynthesis was closely correlated with the contents of starch and its components (amylose and amylopectin) and with the contents of sucrose and soluble sugars. The expression of genes involved in starch biosynthesis mainly affected the accumulation of amylopectin and total starch, and the expressions of most of the 23 genes showed significant correlations (p < 0.05 or p < 0.01) with amylopectin and total starch content in most of the different sampling periods (**Table 4**).

**Table 4:** Correlation analysis between the expression of genes involved in starch biosynthesis and the dynamic accumulation of starch, sucrose, and soluble sugars.

Gene name	Amylopectin content				Amylose content				Total starch content				Sucrose content				Soluble sugar content			
	Days after anthesis				Days after anthesis				Days after anthesis				Days after anthesis				Days after anthesis			
	14 d	18 d	22 d	26 d	14 d	18 d	22 d	26 d	14 d	18 d	22 d	26 d	14 d	18 d	22 d	26 d	14 d	18 d	22 d	26 d
AGPS1-a	−0.619*	−0.889**	0.467	0.738**	−0.709**	−0.511	0.195	0.680*	−0.641*	−0.799**	0.436	0.742**	−0.856**	0.923**	0.469	0.339	−0.588*	0.191	−0.286	−0.736**
AGPS1-b	−0.522	−0.855**	0.587*	0.838**	−0.664*	−0.444	0.457	0.713**	−0.553	−0.750**	0.595*	0.827**	−0.522	0.957**	0.354	0.465	−0.605*	0.109	−0.402	−0.811**
AGPS2	−0.252	0.053	0.876**	0.835**	−0.302	−0.35	0.716**	0.765**	−0.264	−0.096	0.896**	0.839**	0.048	0.004	−0.698*	0.773**	−0.013	−0.528	−0.585*	−0.985**
AGPL1	−0.927**	−0.891**	0.286	0.523	−0.833**	−0.627*	−0.007	0.444	−0.917**	−0.844**	0.239	0.516	−0.939**	0.880**	0.625*	0.096	−0.609*	0.138	−0.168	−0.627*
AGPL2	−0.347	−0.622*	0.861**	0.963**	−0.227	−0.767**	0.335	0.830**	−0.328	−0.713**	0.799**	0.954**	0.031	0.424	−0.23	0.660*	0.078	−0.343	−0.384	−0.694*
SSI	−0.482	−0.927**	0.184	0.635*	−0.506	−0.553	−0.229	0.453	−0.49	−0.841**	0.105	0.604*	−0.752**	0.720**	0.618*	0.187	−0.47	0.311	−0.068	−0.630*
SSIIa	−0.911**	−0.396	0.753**	0.733**	−0.786**	−.577*	0.12	0.505	−0.895**	−0.488	0.661*	0.693*	−0.898**	0.42	−0.09	0.304	−0.533	−0.252	−0.382	−0.658*
SSIIb	0.5	0.0325	−0.976**	0.22	0.402	0.2	−0.654*	0.265	0.486	0.098	−0.966**	0.238	0.134	−0.37	0.586*	0.132	0.011	0.552	0.627*	−0.703*
SSIIc	−0.379	−0.803**	−0.136	0.946**	−0.277	−0.326	−0.301	0.784**	−0.363	−0.670*	−0.18	0.928**	−0.599*	0.886**	0.846**	0.700*	−0.342	0.271	0.099	−0.581*
SSIIIa	−0.937**	−0.744**	0.275	0.816**	−0.806**	−0.332	−0.006	0.686*	−0.920**	−0.632*	0.23	0.804**	−0.757**	0.984**	0.637*	0.404	−0.405	0.093	−0.161	−0.736**
SSIIIb	−0.803**	−0.649*	0.648*	0.707*	−0.646*	−0.43	0.333	0.620*	−0.780**	−0.604*	0.619*	0.703*	−0.693*	0.880**	0.279	0.305	−0.418	−0.391	−0.431	−0.724**
SSIV	−0.979**	−0.743**	−0.436	−0.554	−0.923**	−0.244	−0.655*	−0.481	−0.976**	−0.598*	−0.51	−0.549	−0.858**	0.885**	−0.15	−0.810**	−0.554	0.322	0.757**	0.547
GBSSI	0.104	−0.22	0.955**	0.778**	−0.177	−0.636*	0.498	0.614*	0.052	−0.39	0.914**	0.755**	−0.093	0.029	−0.5	0.751**	−0.27	−0.414	−0.556	−0.362
GBSSII	−0.788**	0.760**	−0.789**	0.26	−0.690*	0.905**	−0.495	0.222	−0.776**	0.859**	−0.774**	0.257	−0.887**	−0.203	0.821**	−0.186	−0.553	−0.144	0.439	−0.379
BEI	0.834**	−0.867**	0.716**	0.840**	0.872**	−0.662*	0.368	0.800**	0.848**	−0.840**	0.684*	0.852**	0.692*	0.889**	0.174	0.48	0.503	−0.13	−0.483	−0.738**
BEIIa	−0.933**	−0.795**	0.899**	0.905**	−0.882**	−0.686*	0.389	0.686*	−0.931**	−0.800**	0.843**	0.872**	−0.966**	0.817**	−0.39	0.594*	−0.653*	−0.19	−0.471	−0.639*
BEIIb	−0.773**	−0.856**	0.22	0.477	−0.649*	−0.583*	0.079	0.404	−0.756**	−0.803**	0.203	0.47	−0.846**	0.929**	0.680*	0.051	−0.506	−0.026	−0.221	−0.593*
BEIII	0.707*	0.764**	−0.118	0.288	0.649*	0.863**	−0.04	0.19	0.702*	0.846**	−0.11	0.27	0.881**	−0.444	0.830**	0.583*	0.600*	0.198	0.035	−0.215
ISA1	−0.580*	−0.068	0.928**	0.927**	−0.652*	−0.407	0.443	0.731**	−0.598*	−0.2	0.879**	0.900**	−0.319	0.154	−0.594*	0.584*	−0.245	−0.578*	−0.49	−0.657*
ISA2	−0.207	−0.476	−0.471	−0.028	−0.21	−0.236	−0.558	−0.075	−0.209	−0.413	−0.52	−0.041	−0.533	0.095	0.886**	−0.473	−0.374	0.754**	0.359	−0.029
PHOH	−0.099	−0.689*	0.629*	0.548	0.074	−0.205	0.073	0.483	−0.067	−0.547	0.546	0.546	−0.304	0.762**	0.680*	0.364	0.006	0.319	−0.257	−0.459
PHOL	−0.282	−0.06	0.836**	0.881**	−0.396	−0.457	0.289	0.685*	−0.305	−0.213	0.768**	0.853**	−0.232	0.820**	0.101	0.057	−0.274	−0.56	−0.361	−0.546
PUL	0.068	−0.630*	0.2	0.764**	0.021	−0.105	−0.077	0.683*	0.06	−0.469	0.151	0.763**	−0.103	0.067	−0.43	0.5	−0.281	0.359	−0.066	−0.745**

Asterisks indicate significant correlations at p< 0.05 (*) and p < 0.01 (**).

### Correlation Analysis of the Activity of Key Starch Biosynthesis Enzymes With the Dynamic Accumulation of Soluble Sugars, Sucrose, and Starch

The enzyme activities during different phases of grain filling were found to be closely related to the dynamic accumulation of starch and sucrose (**Table 5**). The correlations of *SS* activity with the amylopectin and starch content were significant (p < 0.05 or p < 0.01) in different sampling times. The correlations of *SBE* and *AGPase* activities with the amylopectin and starch content were significant (p < 0.01) from 22 to 26 DAA. The correlations of *GBSS* and *SS* activities with the amylopectin and starch content were significant (p < 0.05 or p < 0.01) except for 18 DAA. The *SSS* activity was more closely related to the dynamic accumulation of amylose, amylopectin, and total starch than the other key enzymes. And the activities of starch biosynthetic key enzymes were more closely related to the amylopectin and total starch content than to the amylose content, sucrose content, and soluble sugar content. The correlations between the activities of starch biosynthetic key enzymes and starch accumulation rate were significant (p < 0.05 or p < 0.01) (except for *SBE* and *AGPase* activity at 14 DAA). Which indicated that the activity of starch biosynthetic key enzymes directly influenced starch accumulation in wheat endosperm.

**Table 5:** Correlation analysis between the activities of key starch biosynthesis enzymes and the dynamic accumulation of starch, sucrose, and soluble sugars.

Key enzyme	Days after anthesis	Amylopectin content	Amylose content	Total starch content	Starch accumulation rate	Sucrose content	Soluble sugar content
GBSS	14 d	0.711**	0.828**	0.739**	0.628*	0.719**	0.339
	18 d	0.031	0.056	0.042	0.793**	0.532	−0.576
	22 d	0.830**	0.552	0.821**	0.942**	0.004	−0.604*
	26 d	0.909**	0.790**	0.902**	0.977**	0.555	−0.784**
SSS	14 d	0.880**	0.756**	0.864**	0.689*	0.676*	0.514
	18 d	0.637*	0.749**	0.717**	0.296	−0.006	−0.308
	22 d	0.669*	0.356	0.642*	0.873**	0.258	−0.413
	26 d	0.953**	0.758**	0.927**	0.974**	0.649*	−0.653*
SBE	14 d	0.184	0.310	0.209	0.443	0.516	0.703*
	18 d	−0.305	−0.447	−0.377	0.974**	0.467	−0.433
	22 d	0.914**	0.456	0.870**	0.872**	−0.596*	−0.556
	26 d	0.968**	0.835**	0.959**	0.884**	0.828**	−0.688*
AGP	14 d	−0.098	−0.224	−0.122	0.108	−0.093	−0.311
	18 d	0.274	0.150	0.243	0.890**	0.142	−0.572
	22 d	0.883**	0.382	0.828**	0.994**	−0.184	−0.492
	26 d	0.968**	0.808**	0.951**	0.898**	0.810**	−0.682*
SS	14 d	0−.674*	−0.464	−0.640*	−0.764**	−0.609*	−0.278
	18 d	−0.531	−0.507	−0.553	0.833**	0.700*	−0.284
	22 d	0.925**	0.528	0.895**	0.922**	−0.451	−0.610*
	26 d	0.962**	0.833**	0.953**	0.890**	0.817**	−0.648*

Asterisks indicate significant correlations at p < 0.05 (*) and p < 0.01 (**).

### The Starch Biosynthesis Process in Response to High Temperature and Drought Stress

**FIGURE 6:**
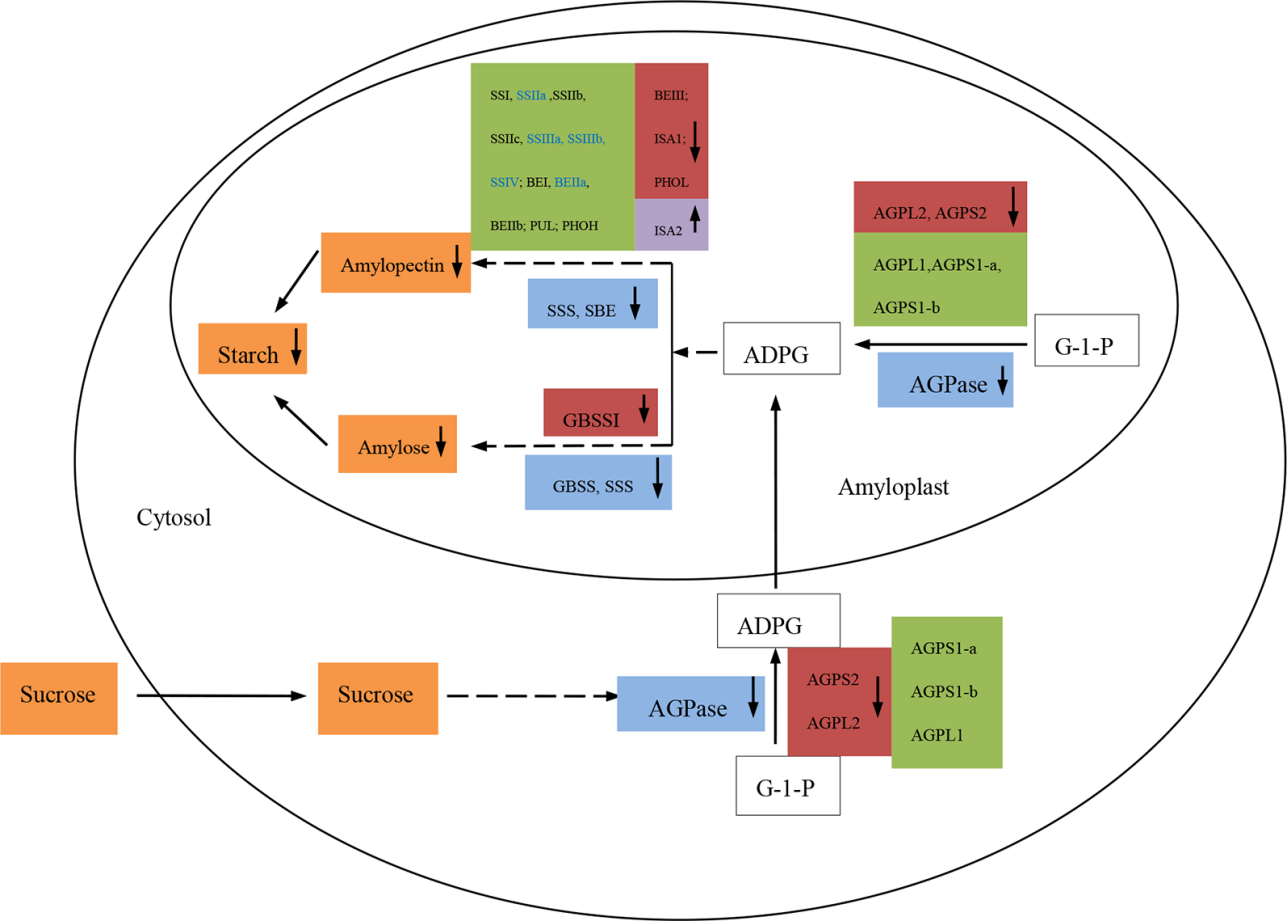
A simplified model of the starch biosynthesis pathway under conditions of high temperature and drought stress. Red boxes: genes in which the expression levels were down-regulated (down arrow) by high temperature (HT), drought stress (DS), and HT+DS treatments. Purple boxes: the only gene (*ISA2*) in which the expression level was up-regulated (up arrow) by HT, DS, and HT+DS treatments. Green boxes: genes in which the expression levels were down-regulated (down arrows) by HT but up-regulated (up arrows) by DS. Genes in which the names are shown in blue were down-regulated (down arrows) by the combined HT+DS treatment. Blue boxes: Key enzymes involved in starch biosynthesis in which the activities were reduced (down arrows).Orange boxes: sugars and starches.

The simplified model of the starch biosynthetic pathway was demonstrated (**Figure 6**, **Supplementary Figure 1**). In response to the adverse environmental stress treatments (HT, DS, and HT+DS), we found that the activities of *AGPase*, *SSS*, GBSS, and *SBE*, all of which are key enzymes associated with the conversion of sucrose to starch, were reduced, and this could explain the decline in starch accumulation. Transcription of the genes encoding *AGPase* isoforms (*AGPS1-a, AGPS1-b, AGPS2, AGPL1,* and *AGPL2*) was reduced by the HT and HT+DS treatments, leading to the repression of the conversion of sucrose from glucose-1-phosphate (*G-1-P*) to *ADP-glucose* (*ADPG*). The expression of all the main genes involved in the conversion of *ADPG* to amylopectin (*SSI, SSIIa, SSIIb, SSIIc, SSIIIa, SSIIIb, SSIV, BEI, BEIIa, BEIIb, PUL, PHOH BEIII, ISA1,* and *PHOL*) was reduced to some extent in response to HT, DS, and HT+DS, although the expression of *ISA2* increased. This led to a significant decrease in the amylopectin content and was also the primary cause of the decrease in total starch content. Under DS, the activities of *AGPase, SSS*, and *SBE* were reduced, although the expression of *AGPS1-a, AGPS1-b, AGPL1, BEI, BEIIa, BEIIb*, and *SS* isozyme genes increased, which implied that starch biosynthetic enzymes did potentially display the coordinated participation of many genes not depend on one or some genes expression. The conversion of sucrose from *G-1-P* to *ADPG* and of *ADPG* to amylopectin was also probably repressed, leading to a significant reduction in the amylopectin and starch content. In the HT, DS, and HT+DS treatments, the decrease in starch accumulation was strongly associated with the change in starch biosynthesis in the wheat grain endosperm, which is attributed to the dynamic change in the activities of key enzymes and the expression profiles of their isozyme genes involved in starch biosynthesis.

## Discussion

### Differences of Genes Encoding Enzymes and Changes of Enzymes Activity Involved in Starch Biosynthesis Under Heat and Drought Stress

There are few reports which describe the expression of genes involved in starch synthesis in wheat under high temperature and DS (Hurkman et al., 2003; Thitisaksakul et al., 2012), although the down-regulation of *PUL* and up-regulation of *SSIIIb* expression in response to HT was previously observed in barley (Mangelsen et al., 2011). High temperature was shown to alter the dynamic changes of the starch biosynthesis, and result in an earlier peak of gene expression in wheat (Li et al., 2017). In the present study, we evaluated all 23 genes encoding enzymes involved in starch biosynthesis in wheat grains and found that their expression profiles were all regulated by HT and DS treatments; in many cases, the genes expression peaked earlier compared with the CK, which was consistent with the results of previous studies. We also found that almost all the 23 genes (apart from *ISA2*) expression was down-regulated by HT, while of the 23 genes expression, 17 was up-regulated by DS. For the HT+DS treatment, almost half-and-half of the 23 genes expression was down-regulated and up-regulated, respectively. High temperature significantly decreased the relative expression of the 23 genes and the negative effect on genes expression was greater than DS.

*SS* was found to be the most sensitive enzyme in starch biosynthesis to drought. The activity of *SS* is highly correlated with starch synthesis rate (Jenner et al., 1993), so that changes in *SS* activity may largely explain the reduced starch content in wheat grains under water stress (Ahmadi and Baker, 2001). Which is a cause of the decrease in the final grain weight (Hawker and Jenner, 1993). The activity of *AGPase* also declines rapidly, especially in response to extreme water deficit, where the loss of *AGPase* activity exceeds that of SS, leading to premature cessation of starch accumulation, and the *GBSS* activity is also affected by drought (Caley et al., 1990; Ahmadi and Baker, 2001). In this study, the DS treatment significantly reduced the activity of *SSS* and *AGPase*, which is consistent with the results of previous studies. We also found that DS caused a decrease in *SBE* activity, but had a weaker effect on *GBSS* activity. In wheat, the *SS* activity is maximal between 20 and 25°C, but at 40°C, 97% of the activity is lost (Keeling et al., 1993). High temperatures significantly reduce the activities of *SS*, *GBSS*, and *SSS*, and also cause a decrease in the relative transcriptional expression of the genes encoding *AGPase*, *SS*, *GBSS*, and *SBE* (Jenner, 1994; Keeling et al., 1994; Hurkman et al., 2003; Zhao et al., 2008). In the present experiment, the HT treatment altered the timing of enzyme activity profiles, leading to earlier peak times compared with the CK. We found that the HT, DS, and HT+DS treatments mainly had negative influences on the activities of *AGPase*, *SSS*, and *GBSS* during the middle and late grain filling stages, but prior to this, heat and DS had less of an effect on these enzymes, or their activities increased to different extents compared with the CK. This could be associated with the adaptive stress response in wheat during a short period of heat or DS. The activities of both SS and SBE were more sensitive to high temperature and DS and showed significant declines during the grain filling stage. The occurrence of gene-specific mRNAs can serve as an indicator of the enzymes potentially involved in starch biosynthesis (Thitisaksakul et al., 2012). There was significant correlation between the starch biosynthetic enzymes activity and the genes expression involved in starch biosynthesis, thus under HT, DS, and HT+DS, the negative effects on starch biosynthetic enzymes activity could be ascribed to the down-regulated expression of the genes involved in starch biosynthesis.

### Starch Biosynthesis in Response to High Temperature and Drought Stress

Four isoforms of starch synthase, *SS I*, *SS II*, *SS III*, and *GBSS I* are expressed in the wheat endosperm during grain filling (Denyer et al., 1995). *SS I* is primarily responsible for the synthesis of the shortest starch chains, and *SS II* and *SS III* contribute to the further extension of the longer chains in amylopectin (Craig et al., 1998; Gao et al., 1998; Umemoto et al., 2002). *GBSS I* is not only responsible for amylose synthesis, but also plays a role in the biosynthesis of extra-long unit chains in amylopectin (Hanashiro et al., 2008). Each isoform contributes differently to the overall starch synthase activity, and gene-specific mRNA levels can act as an indicator of the enzymes that may be present. *SSI* is the major contributor to the total soluble starch synthase activity in wheat endosperm and accounts for almost two-thirds of the enzyme activity (Peng et al., 2001). We found that *SSI* expression showed significant correlations with *SS* activity, while the expression of *SSIIIa* and *SSIIIb* was also closely related to *SS* activity. It indicated that the expression levels of isozyme genes encoding enzymes involved in starch biosynthesis could reflect the activities of the starch biosynthetic enzymes to a certain extent.

Starch biosynthesis and deposition plays the leading role in the process of starch accumulation in wheat grains (Duffus, 1992). In the synthesis process of amylose and amylopectin, the *AGPase* catalyzes the reaction of synthesizing from glucose-1-phosphate to *ADP-glucose*, and the transfer act of the glucosyl moiety of *ADP-glucose* to the non-reducing end of a pre-existing α-(1, 4)-linked glucan primer is catalyzed by *SS*s (Ball et al., 1998; Myers et al., 2000). High temperatures decrease the metabolite levels and enzyme activities associated with the process of converting sucrose to starch (Rijven, 1986; Caley et al., 1990; Jenner, 1991; Hawker and Jenner, 1993; Jenner et al., 1993; Keeling et al., 1994). The inactivation of some enzymes involved in starch biosynthesis inhibits the conversion of sucrose to starch under high temperature conditions, and is the primary cause of the reduction in starch content (Jenner, 1991; Keeling et al., 1994; Yan et al., 2008). Which was also verified in our study. Although another study suggested that starch content is reduced by high temperature because starch accumulation ceases early, shortening the time to achieve the maximum dry grain weight, and not that starch biosynthesis enzymes were repressed (Dupont and Altenbach, 2003). While the shortening time, starch accumulation ceasing early was due to the biochemical and physiological changes (Caley et al., 1990; Ahmadi and Baker, 2001). In our experiment, we found that the activities of *AGPase*, *SSS*, GBSS, and *SBE*, all of which are key enzymes associated with the conversion of sucrose to starch, were reduced, along with the transcription of the genes encoding these enzymes reduced by HT and HT+DS. Thus led to a significant reduction in the amylopectin and starch content in the end. High temperature stress does reduce the conversion of sucrose to starch, but does not affect the sucrose supply for starch synthesis (Bhullar and Jenner, 1986). We found that the stress treatments (DS, HT, and HT+DS) not only repressed the conversion of sucrose to starch, but also led to the decrease of sucrose content in wheat grains.

### Starch Accumulation in Response to Heat and Drought Stress

Temperature and water levels play important roles in starch accumulation. Exposure to high temperatures after flowering reduces the starch content and significantly affects the starch granule size distribution in wheat grains (Hurkman et al., 2003; Zhao et al., 2008). High temperature and DS have been shown to reduce the amylopectin, amylose, and total starch contents (Duffus, 1992; Yan et al., 2008), although the amylose content can increase in response to high temperature, which changes the amylose/amylopectin ratio at maturity (Yan et al., 2008). In this study, HT, DS, and HT+DS all caused decreases in the amylopecin, amylose, and total starch contents, and the ratio of amylose to amylopectin increased due to the more pronounced decrease in the amylopectin content, which supported the previous results of Yan et al. (2008). In our experiments, we also found that wheat plants exposed to the HT, DS, and HT+DS treatments, matured earlier by 8, 4, and 12 days, respectively, compared with the CK. The grain filling process was accelerated and the duration was shortened, which led to a reduction in the starch accumulation time. Also, the HT and DS treatments altered the dynamic changes in starch accumulation with earlier peak times and reduced the starch accumulation rate. The observed decreases in the starch accumulation rate and the early termination of starch accumulation certainly limited starch deposition, leading to a decrease in the starch content. The extent of this reduction would be expected to vary with the timing and duration of HT, the temperature intensity, and plant genotype.

High temperatures after flowering reduce grain starch content (Denyer K. et al., 1994; Chinnusamy and Khanna-Chopra, 2003; Zhao et al., 2008; Labuschagne et al., 2009), and temperatures between 30 and 40°C reduce starch content by 2–33% in wheat (Liu et al., 2011). Which was ascribed to the decline in starch biosynthesis enzyme activity (Keeling et al., 1993; Jenner, 1994; Keeling et al., 1994; Zhao et al., 2008). We found that starch biosynthetic enzyme activities, the expression of their isozyme genes was closely related to the contents of amylopectin, total starch contents, and the dynamic accumulation of starch and sucrose. The activity of starch biosynthetic key enzymes directly influenced starch accumulation in wheat endosperm. Under adverse stress conditions (DS, HT, and HT+DS), the rate and duration of starch accumulation, as well as the starch content was all reduced considerably, which can be attributed to the changes in the expression of genes and the activity of key enzymes involved in starch biosynthesis under adversity stress conditions.

### The Interaction Effect Between High Temperature and Drought Stress

Compared with wheat exposed to either high temperature or DS seperately, the combination of the two stresses mostly reduced wheat growth and led to senescence (Machado and Paulsen, 2001). Short-term exposure (3 and 7 days) to elevated temperature and DS caused additive or multiplicative interactive effects on photosynthetic parameters (namely, the CO_2_ assimilation rate) in wheat (Marcela et al., 2018). Both synergistic or antagonistic effects on wheat grain filling and grain yield can be induced by the HT and DS treatments, with the combination of the two being either more or less severe than either stress alone or the additive effect being more or less severe than expected (Nicolas et al., 1984; Wardlaw, 2002; Shah and Paulsen, 2003). The simultaneous occurrence of high temperature and DS can increase the deleterious effect on wheat yield, such that the combined effect considerably exceeded the simple additive effect of the individual factors (Shah and Paulsen, 2003; Pradhan et al., 2012). In our study, For the HT+DS treatment, the negative effects on starch biosynthesis and accumulation were greater than HT or DS individually, indicating superimposed effect between HT and DS. The combination of HT and DS affected enzyme activities more or less severely than either they alone or their added values, and a significant interaction effect was found in HT and DS treatments. The gene expression profile in the HT+DS treatment was not consistent with the profiles seen for either HT or DS individually, which implied the synergistic or antagonistic interaction effect between HT and DS.

## Conclusions

The results of our study showed that the HT, DS, and HT+DS treatments caused earlier maturation of wheat by 8, 4, and 12 days, respectively, compared with the CK. During the grain filling stage, the dynamic change in the expression of genes and enzyme activity involved in starch biosynthesis in wheat endosperm was all altered, and often appeared to peak values earlier. Of the 23 genes expression, 22 was negatively influenced by HT, while only 6 and 11 was down-regulated by DS and HT+DS, respectively. The expression levels of genes encoding enzymes involved in starch biosynthesis can possibly serve as indicators of the activities of starch biosynthetic enzymes during grain filling. The activity of *AGPase*, *SSS*, *GBSS*, SS, and SBE was significantly decreased by HT, DS, and HT+DS in the end. Under adverse stress conditions, the changes in the expression of genes involved in starch biosynthesis inhibited the activity of starch biosynthetic enzymes associated with the conversion of sucrose to starch. In response to HT, DS, and HT+DS, starch accumulation rate and duration time declined significantly, and accordingly the contents of amylopectin, amylose, and total starch were also significantly reduced. Which was closely related to starch biosynthetic enzyme activities and their isozyme genes expression. In a word, under HT, DS, and HT+DS, the change of enzymes associated with the conversion of sucrose to starch repressed starch accumulation and shortened the time allocated for the grain filling process in wheat, consequently led to the decrease of starch content and grain yield. The trends observed for the HT+DS treatment did not conform to the effects of exposure to HT or DS separately, implying an interaction effect (synergistic or antagonistic) between HT and DS.

## Author Contributions

HL and CW conceived the original research plan. YH and WL performed most of the experiments and analyzed the data. DM and QH supervised the experiments. YH and GM performed the real-time qPCR analysis. HL wrote the article.

## Conflict of Interest

The authors declare that the research was conducted in the absence of any commercial or financial relationships that could be construed as a potential conflict of interest.
