# Supplementary material for: Effects of High Temperature and Drought Stress on the Expression of Gene Encoding Enzymes and the Activity of Key Enzymes Involved in Starch Biosynthesis in Wheat Grains
Source: Front Plant Sci. 2019 Nov 12;10:1414. doi: 10.3389/fpls.2019.01414 (PMC6863091; doi:10.3389/fpls.2019.01414)
Supplement: Supplementary file 1 [file DataSheet_1.pdf]

Table S1. Target genes for analysis of expression profiles and the PCR primer sequences

Isozyme genes; full name	Gene name Abbreviations	Acc. no.	Primer sequence	Amplicon size (bp)
ADP-glucose pyrophosphorylase small subunit I	AGPS1-a	X66080	[F]gggagcaacattggaggtta<br>[R]gcatctgtttctctgtgtgc	224
ADP-glucose pyrophosphorylase small subunit I	AGPS1-b	EU586278	[F]gggagcaacattggaggtta<br>[R]gcatctgtttctctgtgtgc	224
ADP-glucose pyrophosphorylase small subunit II	AGPS2	AY727927	[F]aaccaagaagccagtaccag<br>[R]gagcgtagtccaacaacaga	177
ADP-glucose pyrophosphorylase large subunit I	AGPL1	Z21969	[F]cttgccaccaacaaagtcag<br>[R]atctttgtgttctccccgac	240
ADP-glucose pyrophosphorylase large subunit II	AGPL2	DQ406820	[F]tttgttcagagtcatcggca<br>[R]ggtgtcaacttgcattgctt	171
Starch synthase I	SSI	AJ292521	[F]gaggattccatcgacagcat<br>[R]gcattacaaccatcacacgg	214
Starch synthase IIa	SSIIa	AJ269503	[F]ggggatggaaatctggtgtt<br>[R]tgaagtgttccaggtagtgc	202
Starch synthase IIb	SSIIb	EU333947	[F]acttgcctgaccactacatg<br>[R]gtccagtttccagtcgttct	197
Starch synthase IIc	SSIIc	EU307274	[F]gtgattgctttcatcggacg<br>[R]gagccaacctgacagagaaa	202
Starch synthase IIIa	SSIIIa	AF258608	[F]tggatgcaagtgatgaagct<br>[R]atccgcctcaaatgtgtctt	224
Starch synthase IIIb	SSIIIb	EU333946	[F]agaataagtgcactggtggg<br>[R]tcttgctggctatctcctct	229

Starch synthase IV	SSIV	AY044844	[F]agagagcaactaaacctggc<br>[R]ctggtactggacttgaaccc	163
Granule-bound starch synthase I	GBSSI	AF286320	[F]agtacgagagggtgaggtac<br>[R]tagggtccggagaagtatgg	235
Granule-bound starch synthaseII	GBSSII	AF109395	[F]gcctgtggactctagcatac<br>[R]ctggcattctgtgggtactt	192
Starch branching enzyme I	BEI	Y12320	[F]gtagctctggactctgatgc<br>[R]ctccttcatccttgggcttt	211
Starch branching enzyme IIa	BEIIa	AF286319	[F]ggtggcttgagaagtgtgta<br>[R]tgcccaaactcatttcccat	232
Starch branching enzyme IIb	BEIIb	AY740401	[F]cctacatttgcccttcctgt<br>[R]gcttgatcgtgactttcagc	194
Starch branching enzyme III	BEIII	JQ346193	[F]gaagggcaagaaccagatga<br>[R]agacttgcgtgacgtaactt	150
Starch debranching enzyme Isoamylase I	ISA1	AF548380	[F]atgatcacaacagggacacc<br>[R]cccattccactcagaccaaa	165
Starch debranching enzyme Isoamylase II	ISA2	JX473824	[F] atccgtatgccaaggtgatc<br>[R]ctcgacttgtccttggtgaa	205
Starch debranching enzyme Isoamylase Pullulanase	PUL	EF137375	[F]tattcgcatacacaggaccg<br>[R]atttcagaacctgcgcctta	249
Starch phosphorylase L	PHOL	EU595762	[F]catggggatatggacttcgg<br>[R]tttctcccatcagtgccttc	181
Starch phosphorylase Ha	PHOH	AF275551	[F]ctcattcctggcagtgaact<br>[R]tatccttcctcagaccagca	208

Reference gene (actin)：5'-AAACGAAGGATAGCATGAGGAAGC-3' (F: Forward Primer）

5'-AGCGGTCGAACAACTGGTA-3'(R: Reverse Primer)
