# Supplementary figures and images for: Effects of High Temperature and Drought Stress on the Expression of Gene Encoding Enzymes and the Activity of Key Enzymes Involved in Starch Biosynthesis in Wheat Grains
Source: Front Plant Sci. 2019 Nov 12;10:1414. doi: 10.3389/fpls.2019.01414 (PMC6863091; doi:10.3389/fpls.2019.01414)

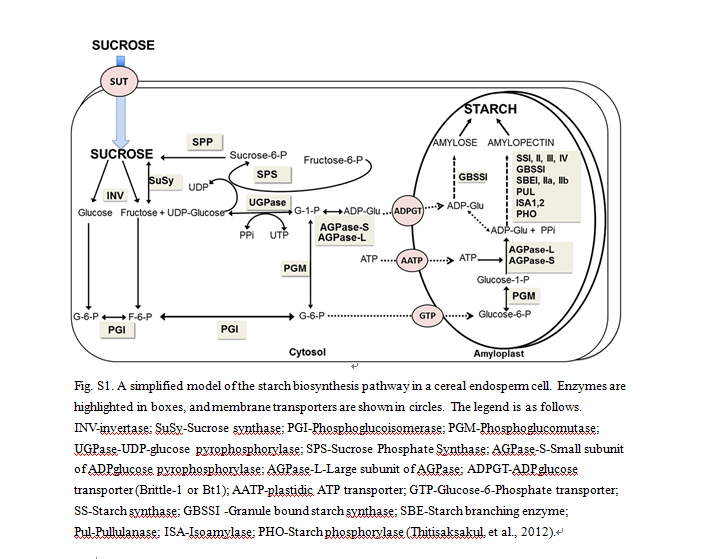

Supplement: Supplementary file 2 [file Image_1.png]
